# KInAs_2_O_7_, a new diarsenate with the TlInAs_2_O_7_ structure type

**DOI:** 10.1107/S2056989017011318

**Published:** 2017-08-04

**Authors:** Karolina Schwendtner, Uwe Kolitsch

**Affiliations:** a TU Wien, Institute for Chemical Technology and Analytics, Division of Structural Chemistry, Getreidemarkt 9/164-SC, 1060 Wien, Austria; b Naturhistorisches Museum Wien, Burgring 7, 1010 Wien, and Universität Wien, Institut für Mineralogie und Kristallographie, Althanstrasse 14, 1090 Wien, Austria

**Keywords:** crystal structure, KInAs_2_O_7_, diarsenate

## Abstract

Potassium indium diarsenate(V) was grown under mild hydro­thermal conditions (*T* = 493 K, 7 d) at a pH value of about 1. It adopts the TlInAs_2_O_7_ structure type (*P*-1, *Z* = 4) and is closely related to the KAlP_2_O_7_ (*P*2_1_/*c*) and RbAlAs_2_O_7_ (*P*-1) structure types. The framework topology of KInAs_2_O_7_ is built of two symmetrically non-equivalent As_2_O_7_ groups which share corners with InO_6_ octa­hedra. The K atoms are located in channels extending along [010].

## Abstract 2

The structure of potassium indium diarsenate(V) is isotypic to TlInAs_2_O_7_ structure type (*P* 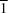, *Z* = 4) and closely related to the KAlP_2_O_7_ (*P*2_1_/*c*) and RbAlAs_2_O_7_ (*P* 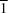) structure types. The framework topology of KInAs_2_O_7_ is built of two symmetrically non-equivalent As_2_O_7_ groups which share corners with InO_6_ octa­hedra. The K atoms are located in channels extending along [010].

## Chemical context   

Metal arsenates often form tetra­hedral–octa­hedral framework structures exhibiting potentially inter­esting properties, such as ion conductivity, ion exchange and catalytic properties (Masquelier *et al.*, 1990 ▸, 1994*a* ▸,*b* ▸, 1995 ▸, 1996 ▸, 1998 ▸; Mesa *et al.*, 2000 ▸; Ouerfelli *et al.*, 2007*a* ▸, 2008 ▸; Pintard-Scrépel *et al.*, 1983 ▸; Rousse *et al.*, 2013 ▸). During a detailed study of the system *M* ^+^–*M* ^3+^–As–O–(H) by hydro­thermal syntheses, a large variety of new compounds and structure types were found (Kolitsch, 2004 ▸; Schwendtner, 2006 ▸; Schwendtner & Kolitsch, 2004*a* ▸,*b* ▸, 2005 ▸, 2007*a* ▸,*b* ▸,*c* ▸,*d* ▸, 2017*a* ▸,*b* ▸). KInAs_2_O_7_ is another example of a microporous metal diarsenate compound forming a tetra­hedral–octa­hedral framework structure.

*M* ^+^ *M* ^3+^As_2_O_7_ compounds crystallize in six known structure types (for a short review, see: Schwendtner & Kolitsch, 2017*b* ▸), some of these diarsenates being also isotypic to diphosphates or disilicates. For several of the structures, the *M* ^+^ cation is the relevant factor that determines which structure type is adopted, while a wide range of different *M* ^3+^ cations are usually accepted. For example, the CaZrSi_2_O_7_ structure type (mineral gittinsite; Roelofsen-Ahl & Peterson, 1989 ▸) is formed by all Li members (and one Na member), with *M* ^3+^ cations ranging from *M* = Al, Ga, Fe to Sc (Schwendtner & Kolitsch, 2007*d* ▸; Wang *et al.*, 1994 ▸). The inter­mediate-sized *M* ^+^ cations Ag^+^ and Na^+^ generally form either of two structure types, the NaInAs_2_O_7_ type (Belam *et al.*, 1997 ▸) or the NaAlAs_2_O_7_ type (Driss & Jouini, 1994 ▸). While the former is only known from the comparatively large *M* ^3+^ cation In^3+^ (Belam *et al.*, 1997 ▸, ICDD-PDF 059-0058; Wohlschlaeger *et al.*, 2007 ▸), the latter is adopted by the smaller *M* ^3+^ representatives (*M* = Al, Fe, Ga) (Ouerfelli *et al.*, 2004 ▸; Schwendtner & Kolitsch, 2017*b* ▸). The larger *M* ^+^ cations (*M* = K, Rb, Cs, Tl, NH_4_) favour three structure types, the stabilities of which seem to be determined mainly by the *M* ^3+^ cations. While the RbAlAs_2_O_7_ type (Boughzala *et al.*, 1993 ▸) is favoured by the smaller cations Al^3+^, Ga^3+^, Cr^3+^ and Fe^3+^ (Boughzala & Jouini, 1992 ▸, 1995 ▸; Bouhassine & Boughzala, 2017 ▸; Lin & Lii, 1996 ▸; Siegfried *et al.*, 2004 ▸; Ouerfelli *et al.*, 2007*a* ▸), the KAlP_2_O_7_ type (Ng & Calvo, 1973 ▸), which is extremely common among *M* ^+^ *M* ^3+^P_2_O_7_ compounds, is favoured by the somewhat larger Sc^3+^ cation (Baran *et al.*, 2006 ▸; Kolitsch, 2004 ▸; Schwendtner & Kolitsch, 2004*a* ▸) and the CsCr member CsCrAs_2_O_7_ (Bouhassine & Boughzala, 2015 ▸). The third type, TlInAs_2_O_7_, is very closely related to the two former types and favoured by the large In^3+^ cation (Schwendtner, 2006 ▸), with also one Fe member (KFeAs_2_O_7_; Ouerfelli *et al.*, 2007*b* ▸). The title compound, KInAs_2_O_7_, is a new member of the latter structure type.

## Structural commentary   

KInAs_2_O_7_ crystallizes in space group *P* 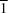 and adopts the TlInAs_2_O_7_ structure type (Schwendtner, 2006 ▸), which is also known for RbInAs_2_O_7_ and NH_4_InAs_2_O_7_ (Schwendtner, 2006 ▸) and KFeAs_2_O_7_ (Ouerfelli *et al.*, 2007*b* ▸) (see comparison in Table 1 ▸).

**Figure 1:**
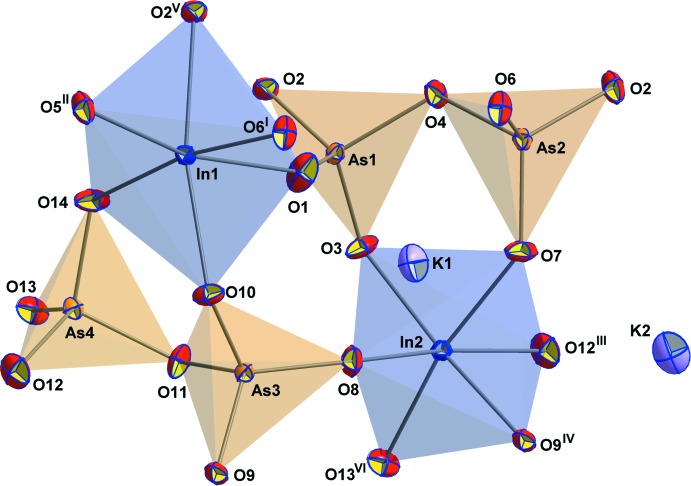
The principal building unit of KInAs_2_O_7_, shown as displacement ellipsoids at the 70% probability level. [Symmetry codes: (i) −*x* + 1, −*y* + 1, −*z* + 1; (ii) *x*, *y* + 1, *z*; (iii) *x*, *y* − 1, *z*; (iv) −*x* + 1, −*y* + 1, −*z*; (v) −*x* + 2, −*y* + 1, −*z* + 1; (vi) −*x* + 2, −*y* + 1, −*z*.]

**Figure 2:**
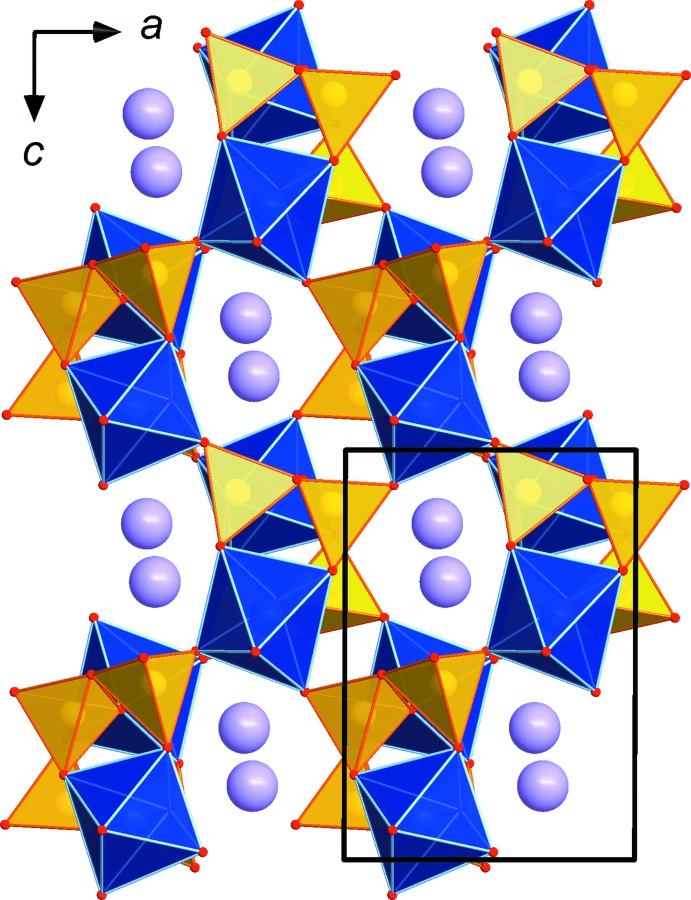
The framework structure of KInAs_2_O_7_, viewed along [010]. The K^+^ cations are hosted in the channels extending along [010]. The unit cell is outlined.

The asymmetric unit contains 22 atoms, all of which lie on general positions. Each InO_6_ octa­hedron shares corners with five different AsO_4_ tetra­hedra, thus creating a framework structure. Two of these connections are to two AsO_4_ tetra­hedra of the same As_2_O_7_ group (see Fig. 1 ▸). The K^+^ cations are situated in small channels extending along [010] (see Fig. 2 ▸) and have irregular coordination spheres, with ten (K1) and seven (K2) O atoms within 3.5 Å.

The AsO_4_ tetra­hedra are strongly distorted, with bond-length distortion (Brown & Shannon, 1973 ▸) ranging from 0.0020 to 0.0024, while the average As—O distances (1.685, 1.687, 1.689 and 1.690 Å for As1–4, respectively, see Table 2 ▸) are typical for As—O bond lengths in diarsenates [average = As—O 1.688 (6) Å; Schwendtner & Kolitsch, 2007*d* ▸]. In addition, the elongated As—O bond lengths to the bridging O atoms (Table 2 ▸), ranging from 1.7485 (16) to 1.7607 (16) Å, are typical for diarsenates [average As—O_bridge_ distance is 1.755 (17); Schwendtner & Kolitsch, 2007*d* ▸]. The As—O_bridge_—As angles are 120.04 (9) and 118.77 (9)°, and therefore very similar to those of the related TlIn, RbIn and NH_4_In compounds (Schwendtner 2006 ▸), but are smaller than the grand mean value in diarsenates, 124 (5)° (Schwendtner & Kolitsch, 2007*d* ▸).

The In1O_6_ octa­hedron is considerably more distorted than the In2-centred octa­hedron. In fact, the In1O_6_ octa­hedron shows the strongest distortion among all of the isotypic In compounds (Schwendtner, 2006 ▸) that are so far known [bond-length distortion (Brown & Shannon, 1973 ▸): 0.0012 (In1), 0.0003 (In2); bond-angle distortion (Robinson *et al.*, 1971 ▸): 66.93 (In1), 20.69 (In2)].

The bond-valence sums, calculated using recently refined parameters (Gagné & Hawthorne, 2015 ▸), amount to 0.94/0.88 (K1/K2), 3.01/2.96 (In1/In2), 5.05/5.03/4.99/4.98 (As1/As2/As3/As4) and 2.00/1.97/1.95/2.08/1.94/1.98/1.99/2.00/2.07/2.02/2.04/1.94/1.89/1.86 (O1–O14) valence units and are thus reasonably close to the theoretical values. As expected, the bridging O4 and O11 ligands are slightly overbonded.

The structure shares a practically identical connectivity with two related structure types, the main difference being differences in space-group symmetry and distortion of the structures. It is most closely related to that of KAlP_2_O_7_ (Ng & Calvo, 1973 ▸), with many of the corresponding Sc-members crystallizing in this structure type. The main difference is a higher space-group symmetry (*P*2_1_/*c*) of the KAlP_2_O_7_ type, which is lost in the In compounds due to the larger ionic radius of In^3+^ and a greater distortion of the structure. The second closely related structure type is that of RbAlAs_2_O_7_ (Boughzala *et al.*, 1993 ▸). Many of the arsenates with large *M* ^+^ and small *M* ^3+^ cations crystallize in this structure type, which is also triclinic (*P* 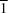), but actually shows higher symmetry, as *Z* is halved and the two distinct positions for the As_2_O_7_ groups, *M* ^3+^O_6_ and *M* ^+^ present in the KAlP_2_O_7_ and TlInAs_2_O_7_ structure types are equivalent in the RbAlAs_2_O_7_ structure type. A more detailed comparison of these three related structure types is given in Schwendtner (2006 ▸).

## Synthesis and crystallization   

KInAs_2_O_7_ was synthesized under mild hydro­thermal conditions at 493 *K* (7 d, autogeneous pressure, slow furnace cooling) using a Teflon-lined stainless steel autoclave with an approximate filling volume of 2 cm^3^. Reagent-grade K_2_CO_3_, In_2_O_3_ and H_3_AsO_4_·5H_2_O were used as starting reagents in approximate volume ratios of *M* ^+^:*M* ^3+^:As of 1:1:2. The vessel was filled with distilled water to about 70% of its inner volume. Initial and final pH was about 1. The reaction products were thoroughly washed with distilled water, filtered and dried at room temperature. KInAs_2_O_7_ grew as thick tabular crystals and was accompanied by about 5 vol.% of K(H_2_O)In(H_1.5_AsO_4_)_2_(H_2_AsO_4_) (Schwendtner & Kolitsch, 2007*c* ▸).

## Refinement   

Crystal data, data collection and structure refinement details are summarized in Table 3 ▸.

The largest residual electron densities in the final difference-Fourier map are below 1 e Å^−3^ and are located close to the In atoms.

## Supplementary Material

Crystal structure: contains datablock(s) I. DOI: 10.1107/S2056989017011318/pk2604sup1.cif

Structure factors: contains datablock(s) I. DOI: 10.1107/S2056989017011318/pk2604Isup2.hkl

CCDC reference: 1565954

Additional supporting information:  crystallographic information; 3D view; checkCIF report

## Figures and Tables

**Table 1:** Comparison of the unit-cell parameters of diarsenates isotypic with KInAs_2_O_7_ and closely related structure types

Compound	*a* (Å)	*b* (Å)	*c* (Å)	α (°)	β (°)	γ (°)	*V* (Å^3^)
**TlInAs_2_O_7_ type** ^1^							
KInAs_2_O_7_	7.712 (2)	8.554 (2)	10.461 (2)	88.58 (3)	89.82 (3)	73.97 (3)	663.1 (3)
RbInAs_2_O_7_^1^	7.845 (2)	8.678 (2)	10.492 (2)	88.85 (3)	89.93 (3)	74.38 (3)	687.5 (3)
TlInAs_2_O_7_^1^	7.827 (2)	8.625 (2)	10.494 (2)	88.83 (3)	89.98 (3)	74.31 (3)	682.1 (3)
(NH_4_)InAs_2_O_7_^1^	7.858 (2)	8.649 (2)	10.515 (2)	88.96 (3)	89.94 (3)	74.34 (3)	688.0 (3)
KFeAs_2_O_7_^2^	7.662 (1)	8.402 (2)	10.100 (3)	89.58 (3)	89.74 (2)	73.61 (2)	623.8 (3)
**KAlP_2_O_7_ type** ^3^							
RbScAs_2_O_7_^4^	7.837 (2)	10.625 (2)	8.778 (2)	90.00	106.45 (3)	90.00	701.0 (3)
TlScAs_2_O_7_^5^	7.814 (2)	10.613 (2)	8.726 (2)	90.00	106.31 (3)	90.00	694.5 (3)
CsCrAs_2_O_7_^6^	7.908 (1)	10.0806 (10)	8.6371 (10)	90.00	105.841 (1)	90.00	662.38 (13)
(NH_4_)ScAs_2_O_7_^7^	7.842 (2)	10.656 (2)	8.765 (2)	90.00	106.81 (3)	90.00	701.1 (3)
**RbAlAs_2_O_7_ type** ^8^							
KGaAs_2_O_7_^9^	6.271 (1)	6.376 (1)	8.169 (1)	96.45 (1)	103.86 (1)	103.87 (1)	302.84 (8)
KAlAs_2_O_7_^10^	6.192 (4)	6.297 (3)	8.106 (1)	96.600 (8)	104.517 (8)	102.864 (7)	293.4
RbAlAs_2_O_7_^8^	6.241 (5)	6.34 (2)	8.233 (5)	96.7 (1)	103.89 (7)	102.6 (1)	303.9
CsAlAs_2_O_7_^11^	6.494 (8)	6.709 (7)	8.360 (8)	97.07 (9)	103.23 (9)	102.62 (8)	340.4
TlAlAs_2_O_7_^11^	6.267 (4)	6.324 (4)	8.168 (8)	97.07 (7)	103.83 (8)	102.99 (8)	300.9
KCr_0.25_Al_0.75_ As_2_O_7_^12^	6.243 (3)	6.349 (3)	8.153 (4)	96.57 (2)	104.45 (3)	103.08 (4)	299.8 (8)
TlFe_0.22_Al_0.78_As_2_O_7_^13^	6.296 (2)	6.397 (2)	8.242 (2)	96.74 (2)	103.78 (2)	102.99 (3)	309.0 (2)
KCrAs_2_O_7_^14^	6.316 (1)	6.420 (1)	8.179 (2)	96.29 (3)	104.27 (3)	103.66 (3)	307.4 (1)

Notes: (1) Schwendtner (2006 ▸), *P* 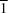, *Z* = 4; (2) Ouerfelli *et al.* (2007*b* ▸), transformed to reduced cell; (3) Ng & Calvo (1973 ▸), *P*2_1_/*c*, *Z* = 4; (4) Schwendtner & Kolitsch (2004*a* ▸); (5) Baran *et al.* (2006 ▸); (6) Bouhassine & Boughzala (2015 ▸); (7) Kolitsch (2004 ▸); (8) Boughzala *et al.* (1993 ▸), *P* 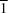, *Z* = 2, transformed to reduced cell; (9) Lin & Lii (1996 ▸); (10) Boughzala & Jouini (1995 ▸); (11) Boughzala & Jouini (1992 ▸), transformed to reduced cell; (12) Bouhassine & Boughzala (2017 ▸); (13) Ouerfelli *et al.* (2007*a* ▸); (14) Siegfried *et al.* (2004 ▸).

**Table 2:** Selected geometric parameters (Å, °)

K1—O6^i^	2.7321 (18)	In2—O9^iv^	2.1243 (16)
K1—O2^ii^	2.7836 (18)	In2—O13^viii^	2.1373 (16)
K1—O8	2.8150 (19)	In2—O3	2.1419 (16)
K1—O6	2.892 (2)	In2—O8	2.1551 (17)
K1—O13^ii^	3.060 (2)	In2—O7	2.1560 (16)
K1—O14^ii^	3.109 (2)	In2—O12^iii^	2.1666 (17)
K1—O10	3.1604 (19)	As1—O1	1.6542 (17)
K1—O1	3.225 (2)	As1—O2	1.6609 (16)
K1—O7	3.289 (2)	As1—O3	1.6761 (16)
K1—O1^i^	3.405 (2)	As1—O4	1.7485 (16)
K2—O10^iii^	2.6849 (19)	As2—O5	1.6592 (16)
K2—O9^iv^	2.7016 (18)	As2—O7	1.6647 (16)
K2—O3^ii^	2.7645 (19)	As2—O6	1.6677 (17)
K2—O7	2.8609 (19)	As2—O4	1.7549 (16)
K2—O12^iv^	2.930 (2)	As3—O8	1.6550 (15)
K2—O9^iii^	3.244 (2)	As3—O9	1.6708 (16)
K2—O5^v^	3.4261 (18)	As3—O10	1.6763 (16)
In1—O5^vi^	2.0946 (17)	As3—O11	1.7538 (16)
In1—O1	2.1036 (17)	As4—O12	1.6579 (17)
In1—O14	2.1502 (17)	As4—O13	1.6697 (16)
In1—O6^i^	2.1618 (16)	As4—O14	1.6727 (16)
In1—O10	2.1643 (16)	As4—O11	1.7607 (16)
In1—O2^vii^	2.1737 (16)		
			
As1—O4—As2	120.04 (9)	As3—O11—As4	118.77 (9)

Symmetry codes: (i) 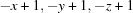; (ii) 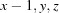; (iii) 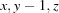; (iv) 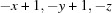; (v) 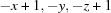; (vi) 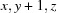; (vii) 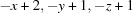; (viii) 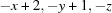.

**Table 3:** Experimental details

Crystal data	
Chemical formula	KInAs_2_O_7_
*M*_r_	415.76
Crystal system, space group	Triclinic, *P* 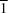
Temperature (K)	293
*a*, *b*, *c* (Å)	7.712 (2), 8.554 (2), 10.461 (2)
α, β, γ (°)	88.58 (3), 89.82 (3), 73.97 (3)
*V* (Å^3^)	663.1 (3)
*Z*	4
Radiation type	Mo *K*α
μ (mm^−1^)	14.09
Crystal size (mm)	0.15 × 0.10 × 0.09

Data collection	
Diffractometer	Nonius KappaCCD single-crystal four-circle
Absorption correction	Multi-scan (*SCALEPACK*; Otwinowski *et al.*, 2003 ▸)
*T*_min_, *T*_max_	0.226, 0.364
No. of measured, independent and observed [*I* > 2σ(*I*)] reflections	11497, 5787, 5467
*R*_int_	0.017
(sin θ/λ)_max_ (Å^−1^)	0.806

Refinement	
*R*[*F* ^2^ > 2σ(*F* ^2^)], *wR*(*F* ^2^), *S*	0.019, 0.043, 1.16
No. of reflections	5787
No. of parameters	200
Δρ_max_, Δρ_min_ (e Å^−3^)	0.91, −0.80

Computer programs: *COLLECT* (Nonius, 2003 ▸), *DENZO* and *SCALEPACK* (Otwinowski *et al.*, 2003 ▸), *SHELXS97* (Sheldrick, 2008 ▸), *SHELXL2016* (Sheldrick, 2015 ▸), *DIAMOND* (Brandenburg, 2005 ▸), *publCIF* (Westrip, 2010 ▸).

## supplementary crystallographic information

### Crystal data

**Table:**

KInAs_2_O_7_	*Z* = 4
*M**_r_* = 415.76	*F*(000) = 760
Triclinic, *P*1	*D*_x_ = 4.165 Mg m^−^^3^
*a* = 7.712 (2) Å	Mo *K*α radiation, λ = 0.71073 Å
*b* = 8.554 (2) Å	Cell parameters from 5760 reflections
*c* = 10.461 (2) Å	θ = 2.5–35.0°
α = 88.58 (3)°	µ = 14.09 mm^−^^1^
β = 89.82 (3)°	*T* = 293 K
γ = 73.97 (3)°	Thick tabular, colourless
*V* = 663.1 (3) Å^3^	0.15 × 0.10 × 0.09 mm

### Data collection

**Table:**

Nonius KappaCCD single-crystal four-circle diffractometer	5467 reflections with *I* > 2σ(*I*)
Radiation source: fine-focus sealed tube	*R*_int_ = 0.017
φ and ω scans	θ_max_ = 35.0°, θ_min_ = 2.5°
Absorption correction: multi-scan (SCALEPACK; Otwinowski *et al.*, 2003)	*h* = −12→12
*T*_min_ = 0.226, *T*_max_ = 0.364	*k* = −13→13
11497 measured reflections	*l* = −16→16
5787 independent reflections	

### Refinement

**Table:**

Refinement on *F*^2^	Primary atom site location: structure-invariant direct methods
Least-squares matrix: full	Secondary atom site location: difference Fourier map
*R*[*F*^2^ > 2σ(*F*^2^)] = 0.019	*w* = 1/[σ^2^(*F*_o_^2^) + (0.0098*P*)^2^ + 1.0091*P*] where *P* = (*F*_o_^2^ + 2*F*_c_^2^)/3
*wR*(*F*^2^) = 0.043	(Δ/σ)_max_ = 0.002
*S* = 1.16	Δρ_max_ = 0.91 e Å^−^^3^
5787 reflections	Δρ_min_ = −0.80 e Å^−^^3^
200 parameters	Extinction correction: SHELXL2016 (Sheldrick, 2015), Fc^*^=kFc[1+0.001xFc^2^λ^3^/sin(2θ)]^-1/4^
0 restraints	Extinction coefficient: 0.00818 (16)

### Special details

**Table:**

Geometry. All esds (except the esd in the dihedral angle between two l.s. planes) are estimated using the full covariance matrix. The cell esds are taken into account individually in the estimation of esds in distances, angles and torsion angles; correlations between esds in cell parameters are only used when they are defined by crystal symmetry. An approximate (isotropic) treatment of cell esds is used for estimating esds involving l.s. planes.

### Fractional atomic coordinates and isotropic or equivalent isotropic displacement parameters (Å^2^)

**Table:**

	*x*	*y*	*z*	*U*_iso_*/*U*_eq_	
K1	0.34522 (8)	0.53997 (7)	0.32187 (5)	0.02034 (10)	
K2	0.31660 (7)	0.05934 (7)	0.17988 (6)	0.02080 (10)	
In1	0.73419 (2)	0.73879 (2)	0.40711 (2)	0.00661 (3)	
In2	0.73416 (2)	0.23051 (2)	0.10125 (2)	0.00664 (3)	
As1	0.94019 (3)	0.32576 (2)	0.35376 (2)	0.00635 (4)	
As2	0.66173 (3)	0.14940 (2)	0.42785 (2)	0.00643 (4)	
As3	0.62526 (3)	0.66936 (2)	0.10396 (2)	0.00657 (4)	
As4	0.95282 (3)	0.79448 (2)	0.13500 (2)	0.00698 (4)	
O1	0.7706 (2)	0.49230 (19)	0.36618 (17)	0.0159 (3)	
O2	1.1358 (2)	0.3418 (2)	0.40953 (15)	0.0119 (3)	
O3	0.9650 (2)	0.2482 (2)	0.20709 (14)	0.0113 (3)	
O4	0.8725 (2)	0.1851 (2)	0.45195 (15)	0.0118 (3)	
O5	0.6925 (2)	−0.03518 (19)	0.49288 (15)	0.0126 (3)	
O6	0.5122 (2)	0.29000 (18)	0.50933 (15)	0.0110 (3)	
O7	0.6078 (2)	0.1721 (2)	0.27322 (14)	0.0140 (3)	
O8	0.5977 (2)	0.48491 (18)	0.12146 (15)	0.0115 (3)	
O9	0.5133 (2)	0.78072 (19)	−0.01871 (14)	0.0114 (3)	
O10	0.5710 (2)	0.78104 (19)	0.23508 (14)	0.0106 (3)	
O11	0.8537 (2)	0.65005 (19)	0.07271 (15)	0.0116 (3)	
O12	0.8338 (2)	0.97121 (19)	0.07213 (16)	0.0149 (3)	
O13	1.1666 (2)	0.7361 (2)	0.08550 (15)	0.0118 (3)	
O14	0.9563 (2)	0.7698 (2)	0.29418 (15)	0.0151 (3)	

### Atomic displacement parameters (Å^2^)

**Table:**

	*U*^11^	*U*^22^	*U*^33^	*U*^12^	*U*^13^	*U*^23^
K1	0.0218 (2)	0.0215 (2)	0.0207 (2)	−0.0113 (2)	0.00072 (19)	0.00221 (18)
K2	0.0139 (2)	0.0180 (2)	0.0283 (3)	−0.00121 (18)	0.00082 (19)	0.00371 (19)
In1	0.00685 (6)	0.00706 (6)	0.00621 (5)	−0.00241 (4)	0.00060 (4)	−0.00022 (4)
In2	0.00646 (6)	0.00762 (6)	0.00602 (5)	−0.00223 (4)	0.00010 (4)	−0.00027 (4)
As1	0.00562 (8)	0.00642 (8)	0.00720 (8)	−0.00194 (6)	−0.00088 (6)	−0.00041 (6)
As2	0.00676 (8)	0.00649 (8)	0.00648 (8)	−0.00255 (6)	0.00150 (6)	−0.00048 (6)
As3	0.00675 (8)	0.00614 (8)	0.00705 (8)	−0.00214 (6)	−0.00117 (6)	−0.00007 (6)
As4	0.00620 (8)	0.00841 (8)	0.00674 (8)	−0.00270 (7)	0.00132 (6)	−0.00005 (6)
O1	0.0121 (7)	0.0076 (6)	0.0253 (8)	0.0021 (5)	−0.0034 (6)	−0.0053 (6)
O2	0.0096 (6)	0.0171 (7)	0.0110 (6)	−0.0072 (6)	−0.0046 (5)	0.0047 (5)
O3	0.0088 (6)	0.0177 (7)	0.0075 (6)	−0.0037 (5)	0.0004 (5)	−0.0050 (5)
O4	0.0083 (6)	0.0151 (7)	0.0138 (7)	−0.0067 (5)	−0.0023 (5)	0.0052 (5)
O5	0.0165 (7)	0.0069 (6)	0.0148 (7)	−0.0040 (5)	0.0026 (6)	0.0002 (5)
O6	0.0095 (6)	0.0095 (6)	0.0146 (7)	−0.0035 (5)	0.0051 (5)	−0.0035 (5)
O7	0.0126 (7)	0.0258 (8)	0.0061 (6)	−0.0096 (6)	−0.0002 (5)	0.0017 (6)
O8	0.0129 (7)	0.0067 (6)	0.0156 (7)	−0.0041 (5)	−0.0011 (5)	0.0003 (5)
O9	0.0119 (6)	0.0129 (7)	0.0111 (6)	−0.0067 (5)	−0.0055 (5)	0.0062 (5)
O10	0.0113 (6)	0.0100 (6)	0.0087 (6)	0.0003 (5)	−0.0021 (5)	−0.0022 (5)
O11	0.0083 (6)	0.0111 (6)	0.0166 (7)	−0.0044 (5)	0.0017 (5)	−0.0043 (5)
O12	0.0167 (7)	0.0070 (6)	0.0189 (7)	0.0004 (6)	−0.0004 (6)	0.0010 (5)
O13	0.0067 (6)	0.0177 (7)	0.0104 (6)	−0.0024 (5)	0.0028 (5)	0.0015 (5)
O14	0.0111 (7)	0.0298 (9)	0.0069 (6)	−0.0100 (6)	0.0011 (5)	0.0010 (6)

### Geometric parameters (Å, º)

**Table:**

K1—O6^i^	2.7321 (18)	In1—O14	2.1502 (17)
K1—O2^ii^	2.7836 (18)	In1—O6^i^	2.1618 (16)
K1—O8	2.8150 (19)	In1—O10	2.1643 (16)
K1—O6	2.892 (2)	In1—O2^vii^	2.1737 (16)
K1—O13^ii^	3.060 (2)	In2—O9^iv^	2.1243 (16)
K1—O14^ii^	3.109 (2)	In2—O13^viii^	2.1373 (16)
K1—O10	3.1604 (19)	In2—O3	2.1419 (16)
K1—O1	3.225 (2)	In2—O8	2.1551 (17)
K1—O7	3.289 (2)	In2—O7	2.1560 (16)
K1—O1^i^	3.405 (2)	In2—O12^iii^	2.1666 (17)
K1—As3	3.5000 (12)	As1—O1	1.6542 (17)
K1—As2	3.6985 (16)	As1—O2	1.6609 (16)
K2—O10^iii^	2.6849 (19)	As1—O3	1.6761 (16)
K2—O9^iv^	2.7016 (18)	As1—O4	1.7485 (16)
K2—O3^ii^	2.7645 (19)	As2—O5	1.6592 (16)
K2—O7	2.8609 (19)	As2—O7	1.6647 (16)
K2—O12^iv^	2.930 (2)	As2—O6	1.6677 (17)
K2—O9^iii^	3.244 (2)	As2—O4	1.7549 (16)
K2—O5^v^	3.4261 (18)	As3—O8	1.6550 (15)
K2—As3^iii^	3.6275 (15)	As3—O9	1.6708 (16)
K2—As1^ii^	3.6671 (15)	As3—O10	1.6763 (16)
K2—As3^iv^	3.8247 (13)	As3—O11	1.7538 (16)
K2—As4^iv^	3.8903 (14)	As4—O12	1.6579 (17)
K2—As2	3.9601 (13)	As4—O13	1.6697 (16)
In1—O5^vi^	2.0946 (17)	As4—O14	1.6727 (16)
In1—O1	2.1036 (17)	As4—O11	1.7607 (16)
			
O6^i^—K1—O2^ii^	120.26 (5)	O2^vii^—In1—K1	114.30 (5)
O6^i^—K1—O8	102.77 (5)	K1^i^—In1—K1	68.45 (3)
O2^ii^—K1—O8	128.48 (5)	O9^iv^—In2—O13^viii^	89.55 (6)
O6^i^—K1—O6	78.11 (5)	O9^iv^—In2—O3	172.63 (6)
O2^ii^—K1—O6	63.78 (5)	O13^viii^—In2—O3	97.43 (6)
O8—K1—O6	102.93 (5)	O9^iv^—In2—O8	84.32 (7)
O6^i^—K1—O13^ii^	114.87 (5)	O13^viii^—In2—O8	93.94 (7)
O2^ii^—K1—O13^ii^	109.47 (5)	O3—In2—O8	92.81 (7)
O8—K1—O13^ii^	71.53 (5)	O9^iv^—In2—O7	81.95 (6)
O6—K1—O13^ii^	166.51 (5)	O13^viii^—In2—O7	170.02 (6)
O6^i^—K1—O14^ii^	100.17 (6)	O3—In2—O7	91.30 (6)
O2^ii^—K1—O14^ii^	77.84 (5)	O8—In2—O7	90.42 (7)
O8—K1—O14^ii^	123.20 (5)	O9^iv^—In2—O12^iii^	87.43 (7)
O6—K1—O14^ii^	132.49 (5)	O13^viii^—In2—O12^iii^	87.02 (7)
O13^ii^—K1—O14^ii^	51.67 (5)	O3—In2—O12^iii^	95.25 (7)
O6^i^—K1—O10	57.03 (5)	O8—In2—O12^iii^	171.69 (6)
O2^ii^—K1—O10	176.42 (5)	O7—In2—O12^iii^	87.39 (7)
O8—K1—O10	55.10 (5)	O9^iv^—In2—K2	39.85 (4)
O6—K1—O10	116.56 (5)	O13^viii^—In2—K2	125.79 (5)
O13^ii^—K1—O10	70.97 (5)	O3—In2—K2	134.41 (5)
O14^ii^—K1—O10	100.08 (5)	O8—In2—K2	97.40 (5)
O6^i^—K1—O1	55.08 (5)	O7—In2—K2	44.56 (5)
O2^ii^—K1—O1	128.24 (5)	O12^iii^—In2—K2	75.47 (5)
O8—K1—O1	56.90 (5)	O9^iv^—In2—K2^ix^	54.43 (5)
O6—K1—O1	65.27 (5)	O13^viii^—In2—K2^ix^	59.83 (5)
O13^ii^—K1—O1	118.16 (5)	O3—In2—K2^ix^	131.59 (5)
O14^ii^—K1—O1	149.48 (5)	O8—In2—K2^ix^	127.99 (5)
O10—K1—O1	52.91 (5)	O7—In2—K2^ix^	110.57 (5)
O6^i^—K1—O7	113.18 (5)	O12^iii^—In2—K2^ix^	46.10 (5)
O2^ii^—K1—O7	77.31 (5)	K2—In2—K2^ix^	71.84 (3)
O8—K1—O7	59.57 (5)	O9^iv^—In2—K1	76.25 (5)
O6—K1—O7	51.60 (5)	O13^viii^—In2—K1	130.97 (5)
O13^ii^—K1—O7	116.67 (5)	O3—In2—K1	97.27 (5)
O14^ii^—K1—O7	145.31 (5)	O8—In2—K1	38.84 (5)
O10—K1—O7	105.77 (5)	O7—In2—K1	51.90 (5)
O1—K1—O7	64.43 (5)	O12^iii^—In2—K1	137.44 (5)
O6^i^—K1—O1^i^	64.20 (5)	K2—In2—K1	66.80 (2)
O2^ii^—K1—O1^i^	56.14 (5)	K2^ix^—In2—K1	130.47 (2)
O8—K1—O1^i^	152.21 (5)	O1—As1—O2	114.49 (9)
O6—K1—O1^i^	51.80 (5)	O1—As1—O3	114.04 (9)
O13^ii^—K1—O1^i^	135.76 (5)	O2—As1—O3	110.79 (8)
O14^ii^—K1—O1^i^	84.23 (5)	O1—As1—O4	102.79 (9)
O10—K1—O1^i^	120.95 (5)	O2—As1—O4	107.73 (8)
O1—K1—O1^i^	97.62 (5)	O3—As1—O4	106.13 (8)
O7—K1—O1^i^	101.37 (5)	O1—As1—K2^x^	152.44 (7)
O6^i^—K1—As3	83.20 (4)	O2—As1—K2^x^	69.60 (6)
O2^ii^—K1—As3	154.87 (4)	O3—As1—K2^x^	45.51 (6)
O8—K1—As3	27.77 (3)	O4—As1—K2^x^	101.41 (6)
O6—K1—As3	118.04 (4)	O5—As2—O7	116.83 (9)
O13^ii^—K1—As3	62.61 (4)	O5—As2—O6	111.77 (8)
O14^ii^—K1—As3	108.68 (4)	O7—As2—O6	109.04 (9)
O10—K1—As3	28.57 (3)	O5—As2—O4	102.19 (8)
O1—K1—As3	55.76 (4)	O7—As2—O4	109.84 (8)
O7—K1—As3	85.32 (4)	O6—As2—O4	106.52 (8)
O1^i^—K1—As3	146.83 (4)	O5—As2—K1	148.49 (6)
O6^i^—K1—As2	91.93 (4)	O7—As2—K1	62.78 (7)
O2^ii^—K1—As2	73.27 (4)	O6—As2—K1	48.98 (6)
O8—K1—As2	78.71 (4)	O4—As2—K1	107.28 (6)
O6—K1—As2	25.79 (3)	O5—As2—K1^i^	111.45 (6)
O13^ii^—K1—As2	143.34 (4)	O7—As2—K1^i^	130.87 (7)
O14^ii^—K1—As2	150.96 (4)	O6—As2—K1^i^	40.86 (6)
O10—K1—As2	108.62 (4)	O4—As2—K1^i^	66.62 (6)
O1—K1—As2	56.71 (4)	K1—As2—K1^i^	71.63 (3)
O7—K1—As2	26.75 (3)	O5—As2—K2	89.90 (7)
O1^i^—K1—As2	77.42 (4)	O7—As2—K2	38.85 (6)
As3—K1—As2	98.85 (3)	O6—As2—K2	96.95 (6)
O10^iii^—K2—O9^iv^	103.29 (6)	O4—As2—K2	146.96 (6)
O10^iii^—K2—O3^ii^	148.68 (5)	K1—As2—K2	71.29 (3)
O9^iv^—K2—O3^ii^	108.00 (6)	K1^i^—As2—K2	136.85 (2)
O10^iii^—K2—O7	77.31 (6)	O8—As3—O9	115.24 (8)
O9^iv^—K2—O7	60.53 (5)	O8—As3—O10	113.16 (8)
O3^ii^—K2—O7	119.83 (6)	O9—As3—O10	107.16 (8)
O10^iii^—K2—O12^iv^	107.93 (6)	O8—As3—O11	108.46 (8)
O9^iv^—K2—O12^iv^	75.74 (5)	O9—As3—O11	105.13 (8)
O3^ii^—K2—O12^iv^	78.86 (6)	O10—As3—O11	107.11 (8)
O7—K2—O12^iv^	135.65 (5)	O8—As3—K1	52.42 (6)
O10^iii^—K2—O9^iii^	53.02 (5)	O9—As3—K1	113.45 (6)
O9^iv^—K2—O9^iii^	77.12 (5)	O10—As3—K1	64.39 (6)
O3^ii^—K2—O9^iii^	133.51 (5)	O11—As3—K1	141.33 (6)
O7—K2—O9^iii^	103.15 (5)	O8—As3—K2^vi^	129.12 (6)
O12^iv^—K2—O9^iii^	57.14 (5)	O9—As3—K2^vi^	63.41 (6)
O10^iii^—K2—O5^v^	76.92 (6)	O10—As3—K2^vi^	43.92 (6)
O9^iv^—K2—O5^v^	131.30 (5)	O11—As3—K2^vi^	121.20 (6)
O3^ii^—K2—O5^v^	83.57 (6)	K1—As3—K2^vi^	80.29 (3)
O7—K2—O5^v^	72.55 (5)	O8—As3—K2^iv^	135.36 (6)
O12^iv^—K2—O5^v^	151.70 (5)	O9—As3—K2^iv^	37.63 (6)
O9^iii^—K2—O5^v^	128.64 (5)	O10—As3—K2^iv^	109.87 (6)
O10^iii^—K2—As3^iii^	25.66 (3)	O11—As3—K2^iv^	68.47 (6)
O9^iv^—K2—As3^iii^	91.84 (5)	K1—As3—K2^iv^	149.98 (2)
O3^ii^—K2—As3^iii^	148.57 (4)	K2^vi^—As3—K2^iv^	77.40 (3)
O7—K2—As3^iii^	90.99 (5)	O12—As4—O13	114.01 (9)
O12^iv^—K2—As3^iii^	82.93 (5)	O12—As4—O14	118.28 (9)
O9^iii^—K2—As3^iii^	27.42 (3)	O13—As4—O14	107.10 (8)
O5^v^—K2—As3^iii^	101.70 (4)	O12—As4—O11	104.77 (8)
O10^iii^—K2—As1^ii^	135.28 (4)	O13—As4—O11	104.59 (8)
O9^iv^—K2—As1^ii^	114.06 (5)	O14—As4—O11	106.99 (8)
O3^ii^—K2—As1^ii^	25.63 (3)	O12—As4—K1^x^	151.68 (6)
O7—K2—As1^ii^	99.90 (5)	O13—As4—K1^x^	53.94 (6)
O12^iv^—K2—As1^ii^	104.49 (4)	O14—As4—K1^x^	55.64 (7)
O9^iii^—K2—As1^ii^	156.93 (3)	O11—As4—K1^x^	103.26 (6)
O5^v^—K2—As1^ii^	60.25 (4)	O12—As4—K2^iv^	43.87 (7)
As3^iii^—K2—As1^ii^	154.03 (2)	O13—As4—K2^iv^	103.15 (6)
O10^iii^—K2—As3^iv^	120.46 (5)	O14—As4—K2^iv^	149.68 (6)
O9^iv^—K2—As3^iv^	22.19 (3)	O11—As4—K2^iv^	66.53 (6)
O3^ii^—K2—As3^iv^	89.60 (5)	K1^x^—As4—K2^iv^	153.30 (2)
O7—K2—As3^iv^	80.16 (4)	As1—O1—In1	137.71 (10)
O12^iv^—K2—As3^iv^	58.83 (4)	As1—O1—K1	129.03 (8)
O9^iii^—K2—As3^iv^	80.57 (4)	In1—O1—K1	93.24 (6)
O5^v^—K2—As3^iv^	143.46 (3)	As1—O1—K1^i^	100.72 (8)
As3^iii^—K2—As3^iv^	102.60 (3)	In1—O1—K1^i^	84.15 (6)
As1^ii^—K2—As3^iv^	102.46 (3)	K1—O1—K1^i^	82.38 (5)
O10^iii^—K2—As4^iv^	130.07 (5)	As1—O2—In1^vii^	129.48 (9)
O9^iv^—K2—As4^iv^	67.39 (4)	As1—O2—K1^x^	129.19 (8)
O3^ii^—K2—As4^iv^	63.75 (4)	In1^vii^—O2—K1^x^	99.93 (6)
O7—K2—As4^iv^	125.94 (4)	As1—O3—In2	120.25 (8)
O12^iv^—K2—As4^iv^	23.09 (3)	As1—O3—K2^x^	108.86 (8)
O9^iii^—K2—As4^iv^	77.51 (4)	In2—O3—K2^x^	126.82 (7)
O5^v^—K2—As4^iv^	147.12 (4)	As1—O4—As2	120.04 (9)
As3^iii^—K2—As4^iv^	104.51 (3)	As2—O5—In1^iii^	130.44 (9)
As1^ii^—K2—As4^iv^	88.12 (3)	As2—O5—K2^v^	116.32 (8)
As3^iv^—K2—As4^iv^	46.153 (19)	In1^iii^—O5—K2^v^	113.24 (6)
O10^iii^—K2—As2	71.17 (4)	As2—O6—In1^i^	125.62 (8)
O9^iv^—K2—As2	81.89 (4)	As2—O6—K1^i^	115.60 (8)
O3^ii^—K2—As2	114.46 (4)	In1^i^—O6—K1^i^	107.02 (6)
O7—K2—As2	21.41 (3)	As2—O6—K1	105.23 (7)
O12^iv^—K2—As2	156.80 (4)	In1^i^—O6—K1	96.99 (6)
O9^iii^—K2—As2	112.01 (4)	K1^i^—O6—K1	101.89 (5)
O5^v^—K2—As2	51.49 (3)	As2—O7—In2	135.63 (9)
As3^iii^—K2—As2	91.82 (3)	As2—O7—K2	119.75 (8)
As1^ii^—K2—As2	90.09 (3)	In2—O7—K2	103.52 (6)
As3^iv^—K2—As2	100.84 (3)	As2—O7—K1	90.47 (8)
As4^iv^—K2—As2	145.27 (2)	In2—O7—K1	97.04 (7)
O5^vi^—In1—O1	166.27 (7)	K2—O7—K1	92.93 (5)
O5^vi^—In1—O14	93.20 (7)	As3—O8—In2	142.75 (9)
O1—In1—O14	96.26 (8)	As3—O8—K1	99.81 (8)
O5^vi^—In1—O6^i^	90.56 (7)	In2—O8—K1	112.46 (7)
O1—In1—O6^i^	81.66 (7)	As3—O9—In2^iv^	127.85 (8)
O14—In1—O6^i^	170.49 (6)	As3—O9—K2^iv^	120.18 (8)
O5^vi^—In1—O10	106.60 (7)	In2^iv^—O9—K2^iv^	109.90 (6)
O1—In1—O10	83.61 (7)	As3—O9—K2^vi^	89.16 (7)
O14—In1—O10	88.56 (6)	In2^iv^—O9—K2^vi^	93.39 (6)
O6^i^—In1—O10	81.99 (6)	K2^iv^—O9—K2^vi^	102.88 (5)
O5^vi^—In1—O2^vii^	80.65 (7)	As3—O10—In1	123.52 (8)
O1—In1—O2^vii^	87.70 (7)	As3—O10—K2^vi^	110.42 (7)
O14—In1—O2^vii^	101.65 (6)	In1—O10—K2^vi^	123.98 (7)
O6^i^—In1—O2^vii^	87.57 (6)	As3—O10—K1	87.04 (6)
O10—In1—O2^vii^	167.27 (6)	In1—O10—K1	93.87 (6)
O5^vi^—In1—K1^i^	103.80 (5)	K2^vi^—O10—K1	103.39 (6)
O1—In1—K1^i^	62.60 (6)	As3—O11—As4	118.77 (9)
O14—In1—K1^i^	138.00 (5)	As4—O12—In2^vi^	145.24 (10)
O6^i^—In1—K1^i^	48.79 (5)	As4—O12—K2^iv^	113.04 (8)
O10—In1—K1^i^	121.39 (5)	In2^vi^—O12—K2^iv^	101.71 (7)
O2^vii^—In1—K1^i^	45.94 (4)	As4—O13—In2^viii^	127.33 (9)
O5^vi^—In1—K1	124.31 (5)	As4—O13—K1^x^	99.88 (7)
O1—In1—K1	54.63 (5)	In2^viii^—O13—K1^x^	132.34 (7)
O14—In1—K1	130.46 (5)	As4—O14—In1	124.72 (9)
O6^i^—In1—K1	41.42 (4)	As4—O14—K1^x^	97.99 (8)
O10—In1—K1	52.98 (5)	In1—O14—K1^x^	122.74 (7)

Symmetry codes: (i) −*x*+1, −*y*+1, −*z*+1; (ii) *x*−1, *y*, *z*; (iii) *x*, *y*−1, *z*; (iv) −*x*+1, −*y*+1, −*z*; (v) −*x*+1, −*y*, −*z*+1; (vi) *x*, *y*+1, *z*; (vii) −*x*+2, −*y*+1, −*z*+1; (viii) −*x*+2, −*y*+1, −*z*; (ix) −*x*+1, −*y*, −*z*; (x) *x*+1, *y*, *z*.
